# Genome-wide association study and targeted metabolomics identifies sex-specific association of CPS1 with coronary artery disease

**DOI:** 10.1038/ncomms10558

**Published:** 2016-01-29

**Authors:** Jaana A. Hartiala, W. H. Wilson Tang, Zeneng Wang, Amanda L. Crow, Alexandre F. R. Stewart, Robert Roberts, Ruth McPherson, Jeanette Erdmann, Christina Willenborg, Stanley L. Hazen, Hooman Allayee

**Affiliations:** 1 Department of Preventive Medicine, USC Keck School of Medicine, Los Angeles, California 90033, USA; 2 Institute for Genetic Medicine, USC Keck School of Medicine, Los Angeles, California 90033, USA; 3 Department of Cardiovascular Medicine, Cleveland Clinic, Cleveland, Ohio 44195, USA; 4 Department of Cellular and Molecular Medicine, Cleveland Clinic, Cleveland, Ohio 44195, USA; 5 John and Jennifer Ruddy Canadian Cardiovascular Genetics Centre, University of Ottawa Heart Institute, Ottawa, Ontario, Canada K1Y 4W7; 6 Institute for Integrative and Experimental Genomics, University of Lübeck, DZHK (German Research Centre for Cardiovascular Research), Partner Site Hamburg/Lübeck/Kiel, University Heart Center Luebeck, 23562 Lübeck, Germany

## Abstract

Metabolites derived from dietary choline and L-carnitine, such as trimethylamine *N*-oxide and betaine, have recently been identified as novel risk factors for atherosclerosis in mice and humans. We sought to identify genetic factors associated with plasma betaine levels and determine their effect on risk of coronary artery disease (CAD). A two-stage genome-wide association study (GWAS) identified two significantly associated loci on chromosomes 2q34 and 5q14.1. The lead variant on 2q24 (rs715) localizes to carbamoyl-phosphate synthase 1 (*CPS1*), which encodes a mitochondrial enzyme that catalyses the first committed reaction and rate-limiting step in the urea cycle. Rs715 is also significantly associated with decreased levels of urea cycle metabolites and increased plasma glycine levels. Notably, rs715 yield a strikingly significant and protective association with decreased risk of CAD in only women. These results suggest that glycine metabolism and/or the urea cycle represent potentially novel sex-specific mechanisms for the development of atherosclerosis.

## Abstract (web summary)

Dietary choline metabolites, such as trimethylamine N-oxide and betaine, have been associated with coronary artery disease (CAD). Here, Hartiala *et al*. identify two genetic loci for betaine levels on chromosomes 2q34 and 5q14.1 and find that the 2q34 locus was also associated with other pathway intermediates, and decreased risk of CAD in women.

## Graphical abstract

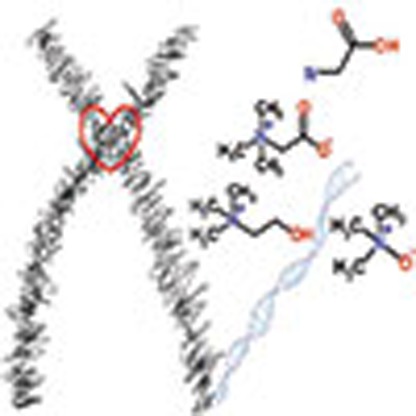

Metabolomics is an emerging field of biomedical research that is based on characterizing the repertoire of small molecules in biological samples. With respect to coronary artery disease (CAD), this has led to the identification of metabolites in several pathways that may serve as novel clinical biomarkers^1^,^2^,^3^. Furthermore, systems genetics analyses that combine metabolic profiles with genomic, transcriptomic, proteomic and clinical data have yielded comprehensive data sets that can potentially be leveraged to identify underlying biological networks that drive disease susceptibility^4^. For example, genome-wide association studies (GWASs) for CAD phenotypes and serum or urinary metabolite levels have provided a catalogue of numerous genetic associations, including variants that are often shown to have pleiotropic effects on multiple metabolites in the same biological pathway^5^. However, these studies have generally been carried out in an unbiased manner, both from a genetics and metabolomics perspective, and efforts are underway to intersect these high-dimensional data to identify pathways causally related to CAD and other diseases.

Another complementary metabolomics approach is to focus on targeted pathways that have been directly implicated in the development of CAD. In this regard, we recently described the role of metabolites derived from dietary choline and L-carnitine on aortic lesion formation in mice and risk of CAD in humans^2^,^6^,^7^. For example, high levels of trimethylamine *N*-oxide (TMAO), which is generated by one or more of the flavin monooxygenase (*FMO*) family of enzymes^8^,^9^ through gut microbiota-dependent metabolism of choline and L-carnitine, have been mechanistically linked to atherosclerosis and increased risk of CAD. Interestingly, naturally occurring DNA variation appears to only play a marginal role in the regulation of TMAO levels, particularly in humans, suggesting that dietary factors and/or gut bacterial composition are more important determinants^10^.

In parallel, dietary choline can also be absorbed by the intestine and oxidized to betaine in the liver (and kidney) via a two-step process involving choline dehydrogenase (*CHDH*) and aldehyde dehydrogenase 7 family member A1 (*ALDH7A1*)^11^,^12^. Although the precise role of TMAO in physiological processes and atherogenesis is not entirely known, both it and *FMO3*, the major enzyme responsible for generating TMAO^8^, have been linked to alterations in cholesterol and sterol metabolism^13^,^14^, as well as inflammatory gene expression^6^,^14^. By comparison, betaine provides one source of methyl groups required for the conversion of homocysteine to methionine, as well the being a methyl donor in folate metabolism^15^. However, the contribution of genetic factors to plasma betaine levels and, by extension, CAD are not well understood. Therefore, the aim of the present study was to integrate targeted metabolomics and GWAS data to identify genetic factors controlling plasma betaine levels and determine their relationship to risk of CAD. These analyses identified two significantly associated loci for betaine levels on chromosomes 2q34 and 5q14.1. The locus on 2q24 was further associated with intermediates in the metabolic pathway leading from choline to urea and also exhibited a protective and strikingly significant female-specific association with risk of CAD.

## Results

### Clinical characteristics of Genebank subjects

Table 1 describes the characteristics of the GeneBank subjects used in this study for whom complete clinical data were available. As expected for a patient population undergoing elective cardiac evaluation by coronary angiography, the majority of subjects were male, had prevalent CAD and were taking lipid-lowering medications (Table 1).

### Two-stage GWAS for plasma betaine levels

**Figure 1:**
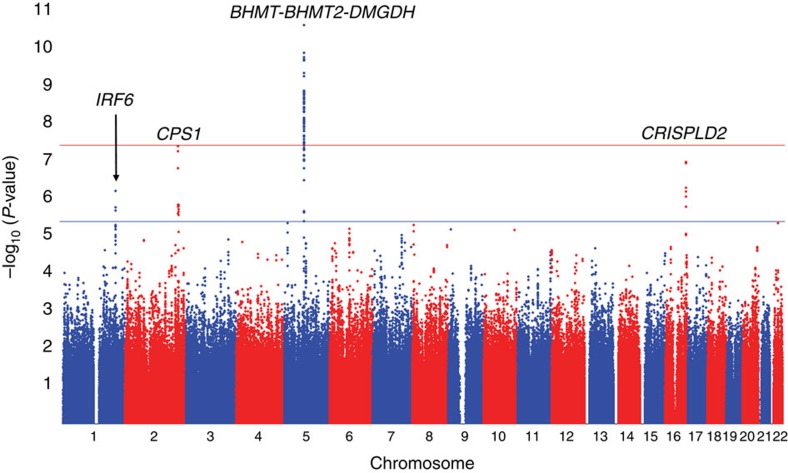
Results of a GWAS for plasma betaine levels in the GeneBank cohort. The Manhattan plot for plasma betaine levels shows four significantly or suggestively associated loci on chromosomes 1q32.2, 2q34 5q14.1 and 16q24.1. The symbols for the genes closest to the lead SNPs are shown in italics and genome-wide thresholds for significant (*P*=5.0 × 10^−8^) and suggestive (*P*=5.0 × 10^−6^) association are indicated by the horizontal red and blue lines, respectively. *P*-values were obtained using linear regression with natural log transformed values and adjustment for age and sex.

**Figure 2:**
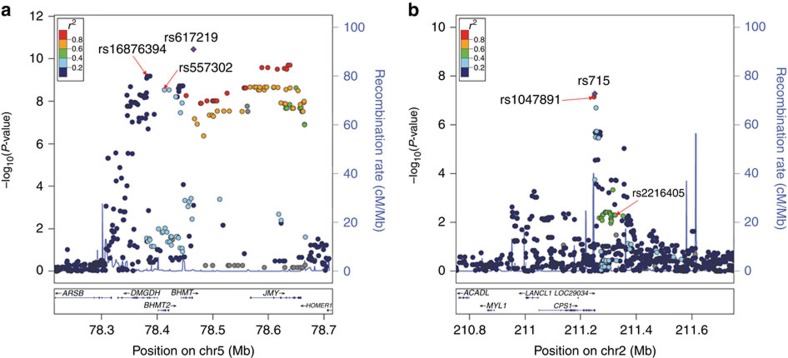
Regional plots for the loci associated with plasma betaine levels. The regions shown for chromosomes 5q14.1 (**a**) and 2q34 (**b**) are centred on the lead SNP (purple diamond) for each respective locus. The degree of LD (*r*^2^) between the lead SNP and other variants in the selected interval is given according the colour-coded legend in the box and genes are indicated in the bottom panel. Rs1047891 was formerly designated as rs7422339.

To identify the genetic determinants of plasma betaine levels, we carried out a two-stage GWAS in sequentially consented and enrolled individuals from GeneBank. In stage 1, ∼2.4 million genotyped and imputed autosomal single-nucleotide polymorphisms (SNPs) were evaluated for association with plasma betaine levels in the first 1,985 subjects, with adjustment for age and sex. The observed genomic control factor in these analyses was 1.007, indicating that the GWAS results are not confounded by underlying population stratification. As shown by the Manhattan plot in Fig. 1, four loci on chromosomes 1q32.2, 2q34, 5q14.1 and 16q24.1 were associated with plasma betaine levels in stage 1 at the significant (*P*<5.0 × 10^−8^) or suggestive (*P*<5.0 × 10^−6^) genome-wide thresholds (Table 2). Multiple SNPs on chromosome 5q14.1 were significantly associated with plasma betaine levels despite not being in strong linkage disequilibrium (LD) with the lead SNP (Fig. 2a), whereas the association signal on chromosome 2q34 was primarily driven by rs715 (Fig. 2b). To determine whether the signals at chromosome 5q14.1 were independent, we performed an analysis conditioned on the lead SNP (rs617219) and identified rs16876394 as still being significantly associated with plasma betaine levels at the genome-wide level (conditioned *P*=8.5 × 10^−9^) as well as a nominal association with rs557302 (*P*=0.002). Aside from rs617219, rs16876394, rs557302 or variants in high LD with these SNPs, there were no other suggestive or significant associations at the chromosome 5q14.1 locus with plasma betaine levels.

In stage 2, we genotyped the lead SNPs at the four identified loci in an additional 1,895 sequential GeneBank subjects for whom plasma betaine levels were measured. The threshold for significance in these replication analyses was set at *P*<8.3 × 10^−3^ based on a Bonferroni correction for testing six SNPs (0.05/6). These analyses failed to replicate the association of rs674433 and rs2641698 with plasma betaine levels (Table 2); therefore, the loci on chromosomes 1q32.2 and 16q24.1 were not given further consideration. However, rs715 on chromosome 2q34 and the three independent SNPs on chromosome 5q14.1 (rs617219, rs16876394 and rs557302) all yielded directionally consistent and statistically significant associations with plasma betaine levels in stage 2, which became even more significant in a combined analysis with all subjects (Table 2).

### Follow-up analyses with chromosome 5q14.1 locus

**Figure 3:**
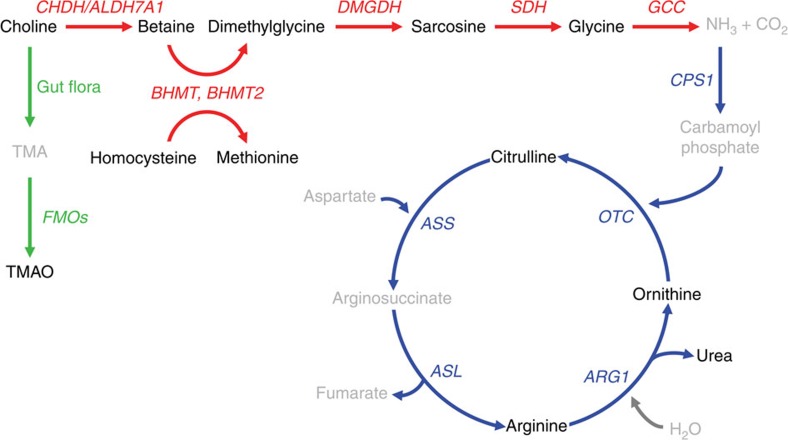
The genes and intermediates of the pathway linking choline metabolism to the urea cycle. One route (green arrows) for the initial catabolism of choline is mediated by intestinal microbes and leads to the formation of trimethylamine (TMA). TMA is efficiently absorbed from the gastrointestinal tract and subsequently oxidized by the liver to form trimethylamine *N*-oxide (TMAO) through reactions catalysed by one or more of the flavin monooxygenase (*FMO*) family of enzymes. Alternatively (red arrows), choline can be oxidized to betaine through reactions catalysed by choline dehydrogenase (*CHDH*) and betaine aldehyde dehydrogenase (*ALDH7A1*). Betaine (also known as trimethylglycine) is demethylated to form dimethylglycine via the betaine-homocysteine *S*-methyltransferase enzymes (*BHMT*, *BHMT2*). This reaction simultaneously converts homocysteine to methionine. Dimethylglycine dehydrogenase (*DMGDH*) subsequently demethylates dimethylglycine to form sarcosine, which is then converted to glycine by sarcosine dehydrogenase (*SDH*) after removal of the remaining methyl group. Glycine is metabolized by a group of enzymes known as the glycine cleavage complex (*GCC*), which is the major route in animals for glycine degradation and the formation of ammonia (NH_3_) and carbon dioxide (CO_2_). NH_3_ is converted to carbamoyl phosphate, which enters the urea cycle (blue arrows) through the rate-limiting reaction catalysed by carbamoyl-phosphate synthase 1 (*CPS1*), or can be converted back to glycine through the *GCC*. Carbamoyl phosphate is metabolized by ornithine transcarbamylase (*OTC*) to form citrulline and subsequently argininosuccinate through a reaction catalysed by argininosuccinate synthetase (*ASS*). This is followed by the formation of L-arginine by arginosuccinate lyase (*ASL*). L-Arginine is used as a substrate for the production of nitric oxide or metabolized by arginase (*ARG1*) to form urea for excretion and ornithine for re-entry back into the cycle. Metabolites that were available for analysis are shown in black, whereas unmeasured metabolites are shown in grey.

The lead SNPs on chromosome 5q14.1 span an ∼200 kb interval containing genes that encode enzymes known to be involved in betaine metabolism, including betaine-homocysteine *S*-methyltransferase (*BHMT*), *BHMT2* and dimethylglycine dehydrogenase (*DMGDH*; Fig. 3). The rs617219 variant maps ∼1,500 bp downstream of *BHMT* and is associated with higher betaine levels, whereas rs16876394 and rs557302 are located in intron 5 of *DMGDH* and intron 4 of *BHTM2*, respectively, and lead to lower betaine levels (Fig. 2a and Table 2). The *BHMT* and *BHMT2* enzymes catalyse the transfer of a methyl group from betaine (also known as trimethylglycine) to homocysteine and simultaneously produce dimethylglycine and methionine, respectively. Dimethylglycine is further metabolized by *DMGDH* through another demethylation reaction to sarcosine, which is subsequently converted to glycine via sarcosine dehydrogenase (*SDH*; Fig. 3).

As chromosome 5q14.1 harbours genes involved in betaine metabolism, we next determined the association of the lead variants at this locus with other analytes in this pathway that were measured in 400 GeneBank subjects selected from stages 1 and 2 who were matched with respect to age, sex and CAD status. At a Bonferroni-corrected threshold of *P*<2.1 × 10^−3^ (0.05/3 SNPs × 8 metabolites), the only significant associations revealed by these analyses were increased and decreased plasma dimethylglycine levels with rs557302 (*P*=6.1 × 10^−4^) and rs617219 (*P*=3.9 × 10^−4^), respectively (Table 3). Furthermore, none of the three chromosome 5q14.1 variants were associated with other pathway-related intermediates, including the pro-atherogenic metabolite TMAO, which is derived from the initial catabolism of choline to trimethylamine (TMA) by gut bacteria, followed by its oxidation to TMAO in the liver by one or more members of the *FMO* family of enzymes^8^ (Table 3 and Fig. 3).

To determine a functional basis for the association of these variants with plasma betaine levels, we searched the publicly available Genotype-Tissue Expression Project database (http://www.gtexportal.org/) for evidence of *cis* expression quantitative trait loci (eQTL) at the chromosome 5q14.1 locus. Of the metabolically relevant tissues available, rs557302 and rs617219, but not rs16876394, yielded *cis* eQTLs for *BHMT*, *BHMT2* or *DMGDH* at varying levels of significance in subcutaneous adipose tissue and skeletal muscle (Supplementary Figs 1–3), but not in the liver or kidney (data not shown).

### Follow-up analyses with chromosome 2q34

The lead SNP on chromosome 2q34 (rs715; T>C) is located in the 3′ untranslated region of the carbamoyl-phosphate synthase 1 gene (*CPS1*), and, in subjects of northern European ancestry, is in near perfect LD (*r*^2^=0.93) with rs1047891 (formerly designated as rs7422339; Fig. 2b). Interestingly, two other previous GWAS identified a different *CPS1* variant (rs2216405) for plasma glycine levels^16^,^17^, which is in moderate LD with rs1047891 and rs715 in subjects of northern European ancestry (*r*^2^∼0.42). In our GWAS analyses, rs2216405 yielded a modest association with plasma betaine levels (*P*=0.005; Fig. 2b) but became non-significant after conditioning on rs715 (conditioned *P*-value=0.39). As a result, we focused our follow-up analyses for the *CPS1* locus on rs715.

*CPS1* encodes a mitochondrial enzyme that catalyses the first committed reaction and rate-limiting step in the urea cycle by generating carbamoyl phosphate from NH_3_ and CO_2_. One of the major routes leading to the formation of NH_3_ and CO_2_ in animals is the degradation of glycine by the glycine cleavage complex (*GCC*; Fig. 3). Therefore, we next determined the association of rs715 with plasma glycine levels and other metabolites leading from choline to the urea cycle in sequential GeneBank subjects and in the subset of 400 individuals in whom betaine-derived analytes were measured. At a Bonferroni-corrected threshold of *P*<4.2 × 10^−3^ (0.05/1 SNP × 12 metabolites), rs715 was significantly associated with increased plasma glycine and decreased citrulline levels (Table 4). Based on previously reported sexually dimorphic effects of rs715 on plasma glycine levels^18^, we also carried out association tests in males and females separately. The association of rs715 with plasma choline, betaine, glycine and citrulline was more pronounced and significant in female subjects, despite comparable or greater numbers of males in these analyses (Table 4). Rs715 also yielded nominally significant female-specific associations with plasma TMAO and other urea cycle metabolites but not with intermediates, such as homocysteine, methionine, dimethylglycine and sarcosine (Table 4). Of note, the significant sexually dimorphic associations of rs715 with plasma choline, betaine and glycine levels were supported by significant interactions with sex (*P*<0.05; Table 4).

### Effect of betaine-associated variants on risk of CAD

To investigate the clinical significance of the loci on chromosomes 2q34 and 5q14.1, we determined whether the identified variants were associated with various CAD phenotypes. In addition to using the subjects in the metabolite analyses described above, we also genotyped additional sequential consenting subjects enrolled in GeneBank with available genomic DNA and clinical phenotype data (total *n*=8,668) for rs715, rs16876394, rs557302 and rs617219. Of these variants, rs715 yielded a protective association (*P*=2.6 × 10^−3^; Table 5) with severe CAD, defined as having ≥50% stenosis in three or more major epicardial arteries, which was significant at a Bonferroni-corrected threshold of testing four SNPs with three CAD phenotypes (0.05/12=4.2 × 10^−3^). By comparison, none of the chromosome 5q14.1 variants were significantly associated with any CAD phenotype (Table 5).

The sexually dimorphic metabolite associations with rs715 suggested to us that the association of this variant with CAD could also differ in men and women and/or be mediated through its effects on metabolite levels. To concurrently test these hypotheses, we carried out sex-stratified multivariate analyses in the subset of 400 age-, sex- and CAD-matched GeneBank subjects for whom genetic, metabolomic and clinical phenotype data were available. In addition, only betaine and glycine were included in the multivariate analyses as these metabolites were associated with rs715 in males and females separately at the Bonferroni-corrected significance threshold and exhibited significant interactions with sex. In a univariate model without the inclusion of metabolite levels, rs715 was associated with decreased risk of CAD in women (*P*=0.04) but not men (*P*=0.54; Supplementary Table 1). The female-specific protective association of rs715 with CAD was still significant in a multivariate model that included betaine levels, but not in models that adjusted for glycine or both metabolites (Supplementary Table 1).

To further evaluate the association of the variants on chromosomes 2q34 and 5q14.1 with prevalent CAD in a larger independent data set, we used the results of a meta-analysis of GWAS data with 22,233 cases and 64,762 controls from the CARDIoGRAM Consortium. Consistent with the results in GeneBank, 715 was associated with decreased risk of CAD (odds ratio (OR)=0.95, 95% confidence interval (CI)=0.92–0.99; *P*=0.01) in CARDIoGRAM (Table 6), whereas none of the variants at the chromosome 5q14.1 locus were associated with CAD (Supplementary Table 2). We also evaluated the association of rs715 in males and females separately using the sex-stratified results from CARDIoGRAM. Notably, the C allele of rs715 exhibited a particularly significant association with decreased risk of CAD in females (OR=0.88, 95% CI=0.83–0.94; *P*=6.3 × 10^−5^) but not in male subjects (Table 6).

## Discussion

Based on recent studies in mice and humans implicating choline-derived metabolites in atherosclerosis, the goal of the present study was to identify the genetic determinants of plasma betaine levels in humans and determine their relationship with risk of CAD. Our GWAS analyses identified and validated two loci on chromosomes 2q34 and 5q14.1 that were significantly associated with plasma betaine levels in a cohort of patients undergoing elective cardiac evaluation. Interestingly, these efforts also identified chromosome 2q34 locus as having a more pronounced effect in women on a cascade of circulating metabolites that lead from choline to urea as well as a strong female-specific association with risk of CAD.

The variants on chromosome 5q14.1 are located in or near several genes (*BHMT*, *BHMT2* and *DMGDH*) that metabolize betaine through a series of demethylation reactions. The association signals were restricted to plasma betaine and dimethylglycine levels and were derived from several independent SNPs with directionally opposite effects. It is possible that our analyses were underpowered to detect associations with other betaine pathway metabolites (that is, methionine or sarcosine) as these were only measured in a subset of ∼400 GeneBank subjects. We suspect that this is unlikely as the Twins UK, KORA and Framingham Heart Study cohorts reported directionally consistent associations between rs16876394, rs557302 and rs617219 with plasma betaine levels but not with other pathway intermediates^19^,^20^. However, these studies did not determine whether betaine-associated variants were associated with risk of CAD. Our analyses further revealed that rs557302 and rs617219 exhibit eQTLs for *BHMT*, *BHMT2* and *DMGDH* in adipose tissue and skeletal muscle, thus providing functional evidence for association of chromosome 5q14.1 with plasma betaine levels. Interestingly, the alleles of rs557302 and rs617219 that increased expression of *BHMT* and *BHMT2*, which would presumably increase betaine catabolism, were unexpectedly associated with higher betaine levels. By contrast, the alleles that increased expression of *DMGDH* were associated with lower dimethylglycine levels, which is a biological effect that is consistent with the direction of the eQTL and metabolite associations. Furthermore, *BHMT*, *BHMT2* and *DMGDH* are predominantly expressed in the liver and kidney but there were no *cis* eQTLs identified for these genes with rs16876394, rs557302, rs617219, or any other chromosome 5q14.1 variants in previously published hepatic gene expression data sets, at least based on the thresholds selected for genome-wide significance in these prior analyses^21^,^22^,^23^,^24^. Thus, additional studies will be required to elucidate the genetic and functional complexity underlying the association of plasma betaine levels with the chromosome 5q14.1 region.

The other locus identified by our GWAS on chromosome 2q34 localizes to *CPS1* and revealed several interesting observations. For example, in both male and female GeneBank subjects, rs715 was primarily associated with decreased betaine and increased glycine levels but the effects were stronger in women. Although these results are consistent with prior studies^18^,^19^,^20^,^25^,^26^,^27^, we additionally demonstrated that rs715 was associated with decreased levels of choline, TMAO and urea cycle products (for example, citrulline) in women as well. With respect to the urea cycle, the strongest effect was on citrulline with increasingly weaker effects on more distal metabolites. Although similar trends were observed in men, the effect sizes on urea cycle metabolites were not as strong and the associations did not reach statistical significance. Moreover, the more prominent associations of rs715 with choline, TMAO, betaine and glycine levels in women were supported by significant statistical evidence for an interaction with sex. Taken together, these data revealed a pattern, at least in women, whereby rs715 was most strongly associated with increased glycine levels and more weakly associated with decreased levels of the most proximal precursors starting at choline and the most distal metabolites in the urea cycle. This pathway also provides at least one plausible unifying mechanism for the pleiotropic associations of *CPS1* with metabolites leading from choline to urea (Fig. 3).

The direction of the associations we and others have detected with the *CPS1* locus suggest that the minor C allele of rs715 and/or other tightly linked variants lead to decreased CPS1 activity/expression. Based on data from the HapMap and 1000 Genomes Projects for subjects of northern European ancestry, rs715 is only in very strong LD with rs1047891 (*r*^2^=0.93), a nonsynonymous Thr1405Asn (ACC>AAC) substitution that together with rs715 comprises a small haplotype block at the 3′ end of *CPS1*. Of these polymorphisms, we speculate that rs1047891 may be the more likely causal SNP as structure-function studies have shown this amino-acid substitution to be located within a CPS1 domain that is important for its allosteric activation by *N*-acetylglutamate (NAG)^28^,^29^. Thus, it is possible that an asparagine at position 1,405 disrupts the interaction between CPS1 and NAG, thereby reducing activation of the enzyme and flux through the urea cycle. Such a notion is supported by the development of severe hyperammonemia and lower plasma citrulline, arginine and ornithine levels in an NAG synthase-deficient mouse model^30^, as well as a previous *in vivo* functional genetics study demonstrating that carriers of rs1047891 had significantly decreased levels of arginine-derived nitric oxide metabolites and agonist-stimulated vasodilation during bradykinin infusion^31^.

Glycine is the downstream product of progressive betaine demethylation and one metabolite that is degraded to NH_3_ for subsequent entry into the urea cycle^32^. Although the functional consequences of rs715 and/or rs1047891 might be predicted to also lead to somewhat elevated blood NH_3_ levels, this genetic effect(s) is apparently not to the same extent as that conferred by loss-of-function alleles resulting in *CPS1* deficiency^33^. For example, ∼10% of subjects in the general population with northern European ancestry are homozygous for the rs715/rs1047891 haplotype but there are no reports that such individuals exhibit overt symptoms of severe hyperammonemia. This observation implies that the minor alleles of the rs715/rs1047891 variants, either alone or in combination, do not decrease CPS1 activity/expression by more than 50%. Nonetheless, the effect of rs715 and/or rs1047891 on CPS1 function may still explain why these variants were associated with increase plasma glycine levels as NH_3_ can be converted back to glycine through bidirectional reactions catalysed by *GCC*^32^. This notion would also be consistent with the hyperglycinemia observed in *CPS1*-deficient patients^33^.

Another major aim of our study was to determine the relationship between betaine-associated loci and risk of CAD. Of the two validated loci for betaine levels, the rare allele of the *CPS1* variant was associated with decreased risk of CAD in GeneBank and CARDIoGRAM. The sex-specific protective effect on CAD risk was also consistent in both studies, with a strikingly significant association observed in only women in CARDIoGRAM. This raises several interesting questions regarding the biological mechanism(s) by which rs715 decreases sex-specific risk of CAD. The most straightforward hypothesis is that the protective effect of rs715 is mediated through one or more of the metabolites/biomarkers that this variant is associated with. For example, the minor alleles of rs715 and rs1047891 have been associated with other CAD-related traits, including increased homocysteine^34^,^35^,^36^,^37^ and creatinine levels^38^, but decreased homoarginine^39^,^40^, high-density lipoprotein^41^, and fibrinogen levels^42^,^43^. However, with the possible exception of the association with fibrinogen levels, which in and of themselves do not appear to be causally related to CAD^43^, the direction of the associations with rs715 and these biomarkers is opposite to what would be expected for a variant that decreases risk of CAD.

Our results would also suggest that choline and betaine are not the likely causal factors for the association of rs715 with CAD as the chromosome 5q14.1 variants had similar effects on betaine levels but were not associated with CAD even in the large CARDIoGRAM Consortium. Furthermore, a recent analysis showed that neither choline nor betaine predicted incident cardiac events when plasma TMAO levels were added to the adjustment model, and that choline and betaine predicted future risk of adverse events only in the context of elevated TMAO levels^44^. Thus, it is possible that the association of rs715 with reduced risk of CAD is mediated through lower TMAO levels as this has been shown to be a pro-atherogenic metabolite in both mice and humans^2^. Other possibilities may be related to the association of rs715 with increased levels of glycine and/or decreased urea cycle metabolites. For example, we previously demonstrated strong clinical associations between increased prevalence of obstructive CAD and elevated citrulline and ornithine levels in GeneBank subjects^45^. Alternatively, the female-specific association of rs715 with CAD may involve increased glycine levels as it was no longer significant after inclusion of this amino acid, but not betaine, in the multivariate model. In this regard, glycine has been shown to have cardioprotective anti-inflammatory properties in endothelial cells, activated macrophages and other leukocytes^46^,^47^, thus providing another possible mechanism for how a genetic factor that increases glycine levels reduces risk of CAD. However, additional studies will still be required to determine whether plasma glycine levels are inversely related to CAD risk and whether glycine metabolism and/or the urea cycle are causally related to the development of atherosclerosis, particularly through sex-specific mechanisms.

In conclusion, the integration of targeted metabolomics with an unbiased genetic screen identified loci on chromosomes 2q34 and 5q14.1 as being associated with plasma levels of analytes related to betaine metabolism. Notably, we also identified a strong association of the chromosome 2q34 locus with decreased risk of CAD in only women. Importantly, this finding represents one of the first female-specific genetic associations for CAD and its magnitude (∼12% decreased risk) was equivalent to the most significantly associated loci identified for CAD to date^48^. Given that such loci still only explain ∼11% of the genetic variation in CAD risk^48^, our results also suggest that a portion of this ‘missing heritability' may reside in sex-specific associations. This highlights the need for future genetics and metabolomics studies to be of sufficient size in order to permit adequately powered analyses in men and women separately.

## Methods

### Study population

The Cleveland Clinic GeneBank study is a single site sample repository generated from consecutive patients undergoing elective diagnostic coronary angiography or elective cardiac computed tomographic angiography with extensive clinical and laboratory characterization and longitudinal observation. Subject recruitment occurred between 2001 and 2007. Ethnicity was self-reported and information regarding demographics, medical history and medication use was obtained by patient interviews and confirmed by chart reviews. All clinical outcome data were verified by source documentation. CAD was defined as adjudicated diagnoses of stable or unstable angina, myocardial infarction (adjudicated definition based on defined electrocardiographic changes or elevated cardiac enzymes), angiographic evidence of ≥50% stenosis in one or more major epicardial vessel, and/or a history of known CAD (documented myocardial infarction, CAD or history of revascularization). The GeneBank Study has been used previously for discovery and replication of novel genes and risk factors for atherosclerotic disease^49^,^50^,^51^,^52^,^53^. The present study was approved by the Institutional Review Boards of the Cleveland Clinic and USC Keck School of Medicine.

### Measurement of plasma metabolites

Metabolite levels in human plasma were quantified using stable isotope dilution high-performance liquid chromatography (HPLC) with online electrospray ionization tandem mass spectrometry on an ABI SCIEX QTRAP 5500 mass spectrometer (Applied Biosystems) interfaced with a Shimatzu HPLC equipped with a phenyl column (4.6 × 2,505 mm^2^, 5 μm RexChrom Phenyl; Regis). Separation was performed using a gradient starting from 10 mM ammonium formate over 0.5 min, then to 5 mM ammonium formate, 25% methanol and 0.1% formic acid over 3 min, held for 8 min, followed by 100% methanol and water washing for 3 min. Metabolites were monitored in multiple reaction monitoring mode using characteristic parent–daughter ion transitions at *m*/*z* ratios for each metabolite. Stable isotope labelled internal standards for each monitored analyte were added to plasma samples before protein precipitation and similarly monitored at the appropriate transitions in multiple reaction monitoring mode. Various concentrations of metabolite standards and a fixed amount of internal standards were spiked into control plasma to prepare the calibration curves for quantification of plasma analytes.

### Genotyping

Genome-wide genotyping of SNPs in humans was performed on the Affymetrix Genome-Wide Human Array 6.0 chip. Using these data and those from 120 phased chromosomes from the HapMap CEU samples (HapMap r22 release, NCBI build 36), genotypes were imputed for untyped autosomal SNPs across the genome using MACH 1.0 software. All imputations were done on the forward (+) strand using 562,554 genotyped SNPs that had passed quality control filters. Analyses with the imputed data set excluded individuals with <90% call rates, and SNPs with Hardy-Weinberg equilibrium *P*-values<0.0001 and call rates <97% or minor allele frequencies <1%. This resulted in 2,421,770 autosomal SNPs that were available for a GWAS analysis in 1,985 GeneBank subjects. Genotyping of individual SNPs selected for replication in stage 2 and association with CAD phenotypes was performed using the TaqMan Allelic Discrimination system (Applied Biosystems). In samples from the GWAS data set that were also genotyped by Taqman, the concordance rate with genotypes obtained from the Affymetrix chip was >99%.

### Statistical analyses

GWAS and individual SNP analyses for plasma betaine levels in GeneBank were carried out using linear regression analyses with natural log transformed values and adjustment for age and sex. To test for association of genetic variants with the presence and severity of CAD in the GeneBank Cohort, we used unconditional logistic or multinomial regression, with adjustment for age, sex, medication use (statins and/or aspirin) and Framingham ATP-III risk score (which includes smoking and diabetes status). To determine whether the association of rs715 with CAD was mediated through its effects on glycine or betaine levels, we carried out multivariate logistic regression with or without the metabolites included in the model. Adjusted ORs with 95% CIs are reported with two-sided *P*-values. All analyses were performed using PLINK 1.07 (ref. 54 or SAS 9.3 (SAS Institute Inc) assuming additive genetic models. For replication, the results of the Coronary Artery Disease Genome-wide Replication And Meta-Analysis (CARDIoGRAM) Consortium were used to determine whether variants identified for plasma betaine levels were associated with CAD. CARDIoGRAM represents a GWAS meta-analysis of CAD comprising a discovery set of 22,233 cases and 64,762 controls, in which logistic regression was first used in each cohort to test for association with CAD using a log-additive model with adjustment for age and sex and taking into account the uncertainty of possibly imputed genotypes. Subsequently, a meta-analysis was performed separately for every SNP from each study that passed the quality control criteria using a fixed effects model with inverse variance weighting or a random effects model, depending on the presence of heterogeneity between studies^55^. The same approach was used to test for association with CAD in males and females separately. The results of these meta-analyses were used to determine whether SNPs at the loci on chromosomes 2q34 and 5q14.1 were associated with CAD.

## Additional information

**How to cite this article:** Hartiala, J. A. *et al*. Genome-wide association study and targeted metabolomics identifies sex-specific association of CPS1 with coronary artery disease. *Nat. Commun*. 7:10558 doi: 10.1038/ncomms10558 (2016).

## Supplementary Material

Supplementary Information Supplementary Figures 1-3, Supplementary Tables 1-2 and Supplementary Note 1

## Figures and Tables

**Table 1:** Clinical characteristics of the GeneBank cohort.

Trait	*n*=8,668
Age (years)	64±11
Male/female	6,092/2,576
Number with CAD at baseline (%)	6,637 (77)
	
*CAD severity*	
0 Vessels (%)	2,396 (28)
1 or 2 Vessels (%)	3,330 (38)
≥3 Vessels (%)	2,942 (34)
BMI (kg m^−2^)	29.6±6.1
Total cholesterol (mg dl^−1^)	170±41
HDL cholesterol (mg dl^−1^)	40±13
LDL cholesterol (mg dl^−1^)	99±34
Triglycerides (mg dl^−1^)	152±110
Number on statin or aspirin therapy (%)	7,185 (83)

BMI, body mass index; CAD, coronary artery disease; HDL, high-density lipoprotein; LDL, low-density lipoprotein

Data are shown as mean±s.d. or numbers of individuals (%).

**Table 2:** Two-stage GWAS results for plasma betaine levels in the GeneBank Cohort.

SNP	Chromosome	Position*	Alleles†	MAF	Stage	0	1	2	*P*-value‡
rs674433	1q32.2	208,031,498	T/G	0.22	Discovery	46.1±17.2(*n*=1,231)	43.2±16.0(*n*=652)	40.8±12.3(*n*=102)	2.2 × 10^−6^
				0.19	Replication	41.8±15.8(*n*=1,119)	40.4±14.9(*n*=477)	43.7±17.7(*n*=78)	0.93
				0.20	Combined	44.0±16.7(*n*=2,350)	42.0±15.6(*n*=1,129)	42.0±14.9(*n*=180)	1.3 × 10^−3^
rs715	2q34	211,251,300	T/C	0.32	Discovery	46.8±16.2(*n*=904)	43.6±17.1(*n*=890)	41.2±15.4(*n*=191)	5.3 × 10^−8^
				0.31	Replication	42.4±16.1(*n*=874)	40.7±14.9(*n*=745)	40.0±14.0(*n*=181)	7.8 × 10^−3^
				0.31	Combined	44.6±16.3(*n*=1,778)	42.3±16.2(*n*=1,635)	40.7±14.7(*n*=372)	1.0 × 10^−8^
rs16876394	5q14.1	78,382,525	T/C	0.10	Discovery	45.9±17.0 (*n*=1,598)	40.6±14.1 (*n*=372)	43.0±14.0 (*n*=15)	6.6 × 10^−10^
				0.10	Replication	42.0±15.7 (*n*=1,350)	39.3±13.8 (*n*=324)	34.9±12.9 (*n*=15)	5.0 × 10^−4^
				0.10	Combined	44.1±16.5 (*n*=2,948)	40.0±14.0 (*n*=696)	38.9±13.9 (*n*=30)	7.2 × 10^−12^
rs557302	5q14.1	78,413,090	G/A	0.49	Discovery	46.9±17.3 (*n*=525)	45.5±16.9 (*n*=993)	41.2±14.8 (*n*=467)	3.0 × 10^−9^
				0.48	Replication	43.2±16.5 (*n*=491)	41.2±14.6 (*n*=935)	39.7±15.4 (*n*=418)	9.3 × 10^−4^
				0.48	Combined	45.2±17.0 (*n*=1,016)	43.4±16.0 (*n*=1,928)	40.5±15.1 (*n*=885)	8.2 × 10^−11^
rs617219	5q14.1	78,465,350	A/C	0.37	Discovery	42.2±15.4(*n*=796)	46.1±17.2(*n*=904)	48.4±17.3(*n*=278)	3.6 × 10^−11^
				0.37	Replication	40.0±15.0(*n*=720)	42.2±15.4(*n*=862)	43.2±16.0(*n*=256)	3.3 × 10^−4^
				0.37	Combined	41.2±15.2(*n*=1,516)	44.2±16.5(*n*=1,766)	46.0±16.9(*n*=534)	7.2 × 10^−13^
rs2641698	16q24.1	83,443,531	C/T	0.33	Discovery	46.9±17.3(*n*=907)	43.3±15.9(*n*=838)	42.6±15.9(*n*=240)	1.4 × 10^−7^
				0.34	Replication	41.5±16.0(*n*=737)	41.6±15.2(*n*=675)	40.8±15.5(*n*=217)	0.91
				0.34	Combined	44.5±16.9(*n*=1,644)	42.5±15.6(*n*=1,513)	41.7±15.7(*n*=457)	1.8 × 10^−4^

MAF, minor allele frequency; SNP, single-nucleotide polymorphism.

Data are shown as mean plasma betaine levels (μM)±s.d. as a function of carrying 0, 1 or 2 copies of the minor alleles for lead GWAS SNPs. Associations were considered significant in the replication analyses at a Bonferroni-corrected threshold of *P*<8.3x10^−3^ (0.05/6 SNPs).

^*^Base pair positions on are given according to NCBI build 36.1 (hg18) of the reference human genome sequence.

^†^Alleles shown as major/minor allele.

^‡^*P*-values were obtained using linear regression with natural log transformed values and adjustment for age and sex.

**Table 3:** Association of chromosome 5q14.1 locus with choline pathway metabolites.

Metabolite	rs16876394		rs557302		rs617219	
	Beta±s.e.(*n*)	*P*-value	Beta±s.e.(*n*)	*P*-value	Beta±s.e.(*n*)	*P*-value
Choline (μM)	−0.004±0.013(*n*=3,580)	0.74	−0.008±0.008(*n*=3733)	0.32	0.001±0.008(*n*=3720)	0.90
TMAO (μM)	−0.011±0.030(*n*=3,659)	0.72	−0.006±0.080(*n*=3814)	0.74	−0.012±0.018(*n*=3802)	0.52
Betaine (μM)	−0.093±0.014(*n*=3,674)	**7.2 × 10**^**−12**^	−0.052±0.008(*n*=3829)	**8.2 × 10**^**−11**^	0.059±0.008(*n*=3816)	**7.2x10**^**−13**^
Homocysteine (μM)	−0.017±0.017(*n*=1,964)	0.31	−0.011±0.010(*n*=1999)	0.26	−0.008±0.010(*n*=1997)	0.44
Methionine (μM)	0.069±0.028(*n*=398)	0.02	0.021±0.018(*n*=399)	0.25	0.013±0.019(*n*=397)	0.50
Dimethylglycine (μM)	0.064±0.043(*n*=396)	0.14	0.091±0.027(*n*=397)	**6.1 × 10**^**−4**^	−0.103±0.029(*n*=395)	**3.9 × 10**^**−4**^
Sarcosine (μM)	0.052±0.051(*n*=398)	0.31	0.053±0.032(*n*=399)	0.10	−0.023±0.035(*n*=397)	0.50
Glycine (μM)	0.038±0.034(*n*=398)	0.27	0.030±0.021(*n*=399)	0.16	−0.003±0.023(*n*=397)	0.89

TMAO, trimethylamine *N*-oxide.

Betas±s.e. and *P*-values for the minor alleles of rs16876394 (C), rs557302 (A), and rs617219 (C) were obtained by linear regression using natural log transformed values, assuming an additive genetic model, and with adjustment for age and sex. Significant associations at a Bonferroni-corrected threshold of *P*<2.1 × 10^-3^ (0.05/3 SNPs × 8 metabolites) are highlighted in bold.

**Table 4:** Association of chromosome 2q34 locus (rs715) with choline pathway and urea cycle metabolites.

Metabolite	Combined			Females			Males			
	*n*	Beta±s.e.	*P*-value	*n*	Beta±s.e.	*P*-value	*N*	Beta±s.e.	*P*-value	*P*-interaction
Choline (μM)	3,695	−0.022±0.008	6.9 × 10^−3^	1,292	−0.053±0.015	**4.8** × **10**^**−4**^	2,403	−0.006±0.010	0.54	7.1 × 10^−3^
TMAO (μM)	3,770	−0.032±0.019	0.1	1,316	−0.089±0.033	7.0 × 10^−3^	2,454	−0.002±0.024	0.93	0.03
Betaine (μM)	3,785	−0.050±0.009	**1.0** × **10**^**−8**^	1,321	−0.078±0.016	**1.7** × **10**^**−6**^	2,464	−0.034±0.010	**5.9** × **10**^**−4**^	0.02
Homocysteine (μM)	1,955	0.005±0.011	0.63	784	0.018±0.018	0.3	1,171	−0.003±0.013	0.82	0.33
Methionine (μM)	399	−0.012±0.019	0.54	199	−0.010±0.026	0.7	200	−0.014±0.028	0.61	0.9
Dimethylglycine (μM)	397	−0.015±0.029	0.6	198	−0.081±0.045	0.08	199	0.038±0.037	0.3	0.04
Sarcosine (μM)	399	0.008±0.034	0.82	199	0.041±0.054	0.45	200	−0.020±0.044	0.66	0.38
Glycine (μM)	399	0.133±0.022	**1.0** × **10**^**−9**^	199	0.181±0.034	**1.2** × **10**^**−7**^	200	0.094±0.028	**6.7** × **10**^**−4**^	0.049
Citrulline (μM)	944	−0.098±0.026	**1.3** × **10**^**−4**^	497	−0.139±0.038	**2.2** × **10**^**−4**^	447	−0.051±0.034	0.13	0.08
Arginine (μM)	946	−0.038±0.016	0.01	498	−0.042±0.021	0.048	448	−0.028±0.022	0.2	0.59
Ornithine (μM)	942	−0.062±0.024	9.3 × 10^−3^	497	−0.088±0.034	0.01	445	−0.028±0.033	0.37	0.18
Urea (mg dl^−1^)	4,813	−0.015±0.008	0.06	1,577	−0.037±0.015	0.02	3,236	−0.004±0.009	0.66	0.046

TMAO, trimethylamine *N*-oxide.

Betas±s.e. and *P*-values are shown for the C allele of rs715 using linear regression and assuming an additive genetic model with adjustment for age and sex. Stratified analyses for males and females were adjusted only for age. Analyses for all traits used natural log transformed values. Metabolites are listed from most proximal to distal in the pathway leading from choline to the urea cycle. Significant associations at a Bonferroni-corrected threshold of *P*<4.2x10^-3^ (0.05/1 SNP × 12 metabolites) in females, males or both sexes combined are highlighted in bold. Interactions with sex were considered significant at *P*<0.05.

**Table 5:** Association of loci on chromosomes 2q34 and 5q14.1 with risk of CAD in the GeneBank Cohort.

SNP (locus)	OR (95% CI)				
rs715 (2q34)	*n*	TT	TC	CC	*P*-value
CAD^−^/CAD^+^	1,966/6,507	1	0.97 (0.86–1.09)	0.83 (0.69–1.01)	0.10
No CAD/mild CAD	2,324/3,257	1	0.97 (0.86–1.10)	0.84 (0.69–1.02)	0.12
No CAD/severe CAD	2,324/2,892	1	0.94 (0.83–1.07)	0.68 (0.55–0.85)	**2.6 × 10**^**−3**^
					
**rs16876394 (5q14.1)**	**n**	**TT**	**TC**	**CC**	***P*-value**
CAD^−^/CAD^+^	1,853/6,131	1	0.98 (0.84–1.13)	0.72 (0.40–1.30)	0.46
No CAD/mild CAD	2,195/3,090	1	1.08 (0.93–1.25)	0.89 (0.48–1.66)	0.44
No CAD/severe CAD	2,195/2,699	1	0.99 (0.84–1.16)	0.85 (0.43–1.65)	0.74
					
**rs557302 (5q14.1)**	**n**	**GG**	**GA**	**AA**	***P*-value**
CAD^−^/CAD^+^	1,983/6,568	1	1.06 (0.93–1.21)	1.00 (0.85–1.17)	0.97
No CAD/mild CAD	2,346/3,291	1	1.04 (0.91–1.19)	1.01 (0.86–1.18)	0.90
No CAD/severe CAD	2,346/2,914	1	1.11 (0.96–1.28)	1.04 (0.88–1.24)	0.60
					
**rs617219 (5q14.1)**	**n**	**AA**	**AC**	**CC**	***P*-value**
CAD^−^/CAD^+^	1,992/6,510	1	1.06 (0.94–1.20)	1.05 (0.88–1.26)	0.41
No CAD/mild CAD	2,345/3,260	1	1.04 (0.92–1.17)	1.11 (0.92–1.32)	0.27
No CAD/severe CAD	2,345/2,897	1	1.10 (0.96–1.25)	1.11 (0.91–1.35)	0.17

CAD, coronary artery disease; CI, confidence interval; OR, odds ratio; SNP, single-nucleotide polymorphism.

CAD severity was defined as having≥50% stenosis in 1 or 2 (mild) or≥3 (severe) major epicardial arteries at the time of cardiac evaluation. OR with 95% CI were obtained from logistic or multinomial regression adjusted for age, sex, medication use (statins or aspirin) and Framingham ATP-III risk score. Significant associations at a Bonferroni-corrected threshold of *P*<4.2x10^−3^ (0.05/4 SNPs × 3 phenotypes) are highlighted in bold.

**Table 6:** Association of chromosome 2q34 Locus (rs715) with Risk of CAD in the CARDIoGRAM Consortium.

Group	*n*	Effect allele frequency	OR (95% CI)	*P*–value
Females	26,905	0.30	0.88 (0.83–0.94)	**6.3 × 10^−5^**
Males	26,772	0.31	1.00 (0.96–1.05)	0.95
Combined	53,667	0.31	0.95 (0.92–0.99)	0.01

CAD, coronary artery disease; CI, confidence interval; OR, odds ratio; SNP, single-nucleotide polymorphism.

Association results are shown for the C allele of rs715. Analyses with all subjects were adjusted for age and sex, whereas sex-stratified analyses were only adjusted for age.
