# Supplementary material for: Genome-wide association study and targeted metabolomics identifies sex-specific association of CPS1 with coronary artery disease
Source: Nat Commun. 2016 Jan 29;7:10558. doi: 10.1038/ncomms10558 (PMC4740183; doi:10.1038/ncomms10558)
Supplement: Supplementary Information — Supplementary Figures 1-3, Supplementary Tables 1-2 and Supplementary Note 1 [file ncomms10558-s1.pdf]

Supplemental Figure 1

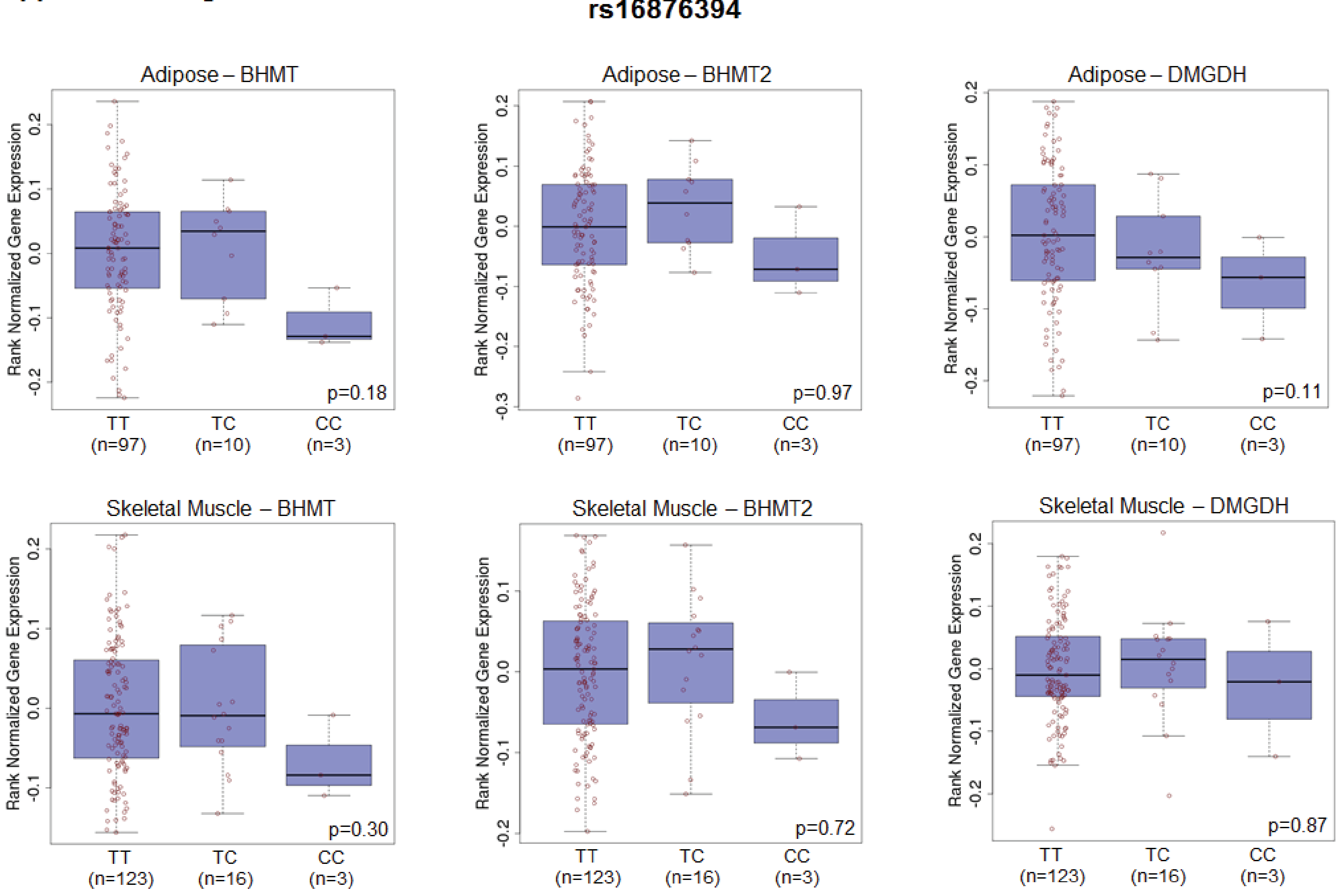

Supplemental Figure 1. *Cis* eQTLs for *BHMT*, *BHMT2*, and *DMGDH* with rs16876394 at the Chromosome 5q14.1 Locus. There were no significant eQTLs in either subcutaneous adipose tissue or skeletal muscle with rs16876394. Data were taken from the publicly available Genotype-Tissue Expression Project database (http://www.gtexportal.org/).

Supplemental Figure 2

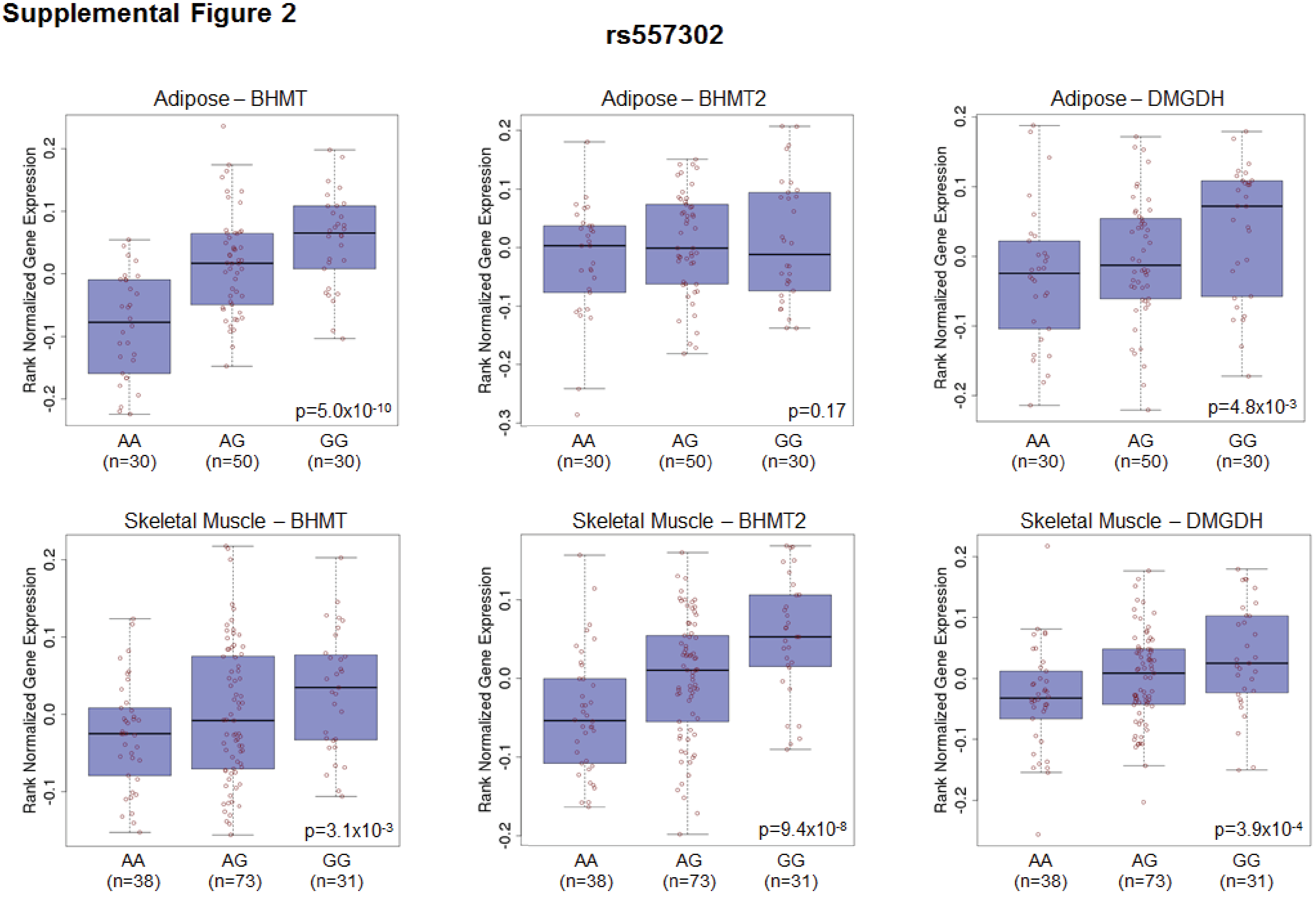

Supplemental Figure 2. ***Cis*** **eQTLs for** ***BHMT*****,** ***BHMT2*****, and** ***DMGDH*** **with rs557302 at the Chromosome 5q14.1 Locus.** Rs557302 yielded eQTLs for *BHMT*, *BHMT2*, or *DMGDH* at varying levels of significance in subcutaneous adipose tissue or skeletal muscle. Data were taken from the publicly available Genotype-Tissue Expression Project database (http://www.gtexportal.org/).

Supplemental Figure 3

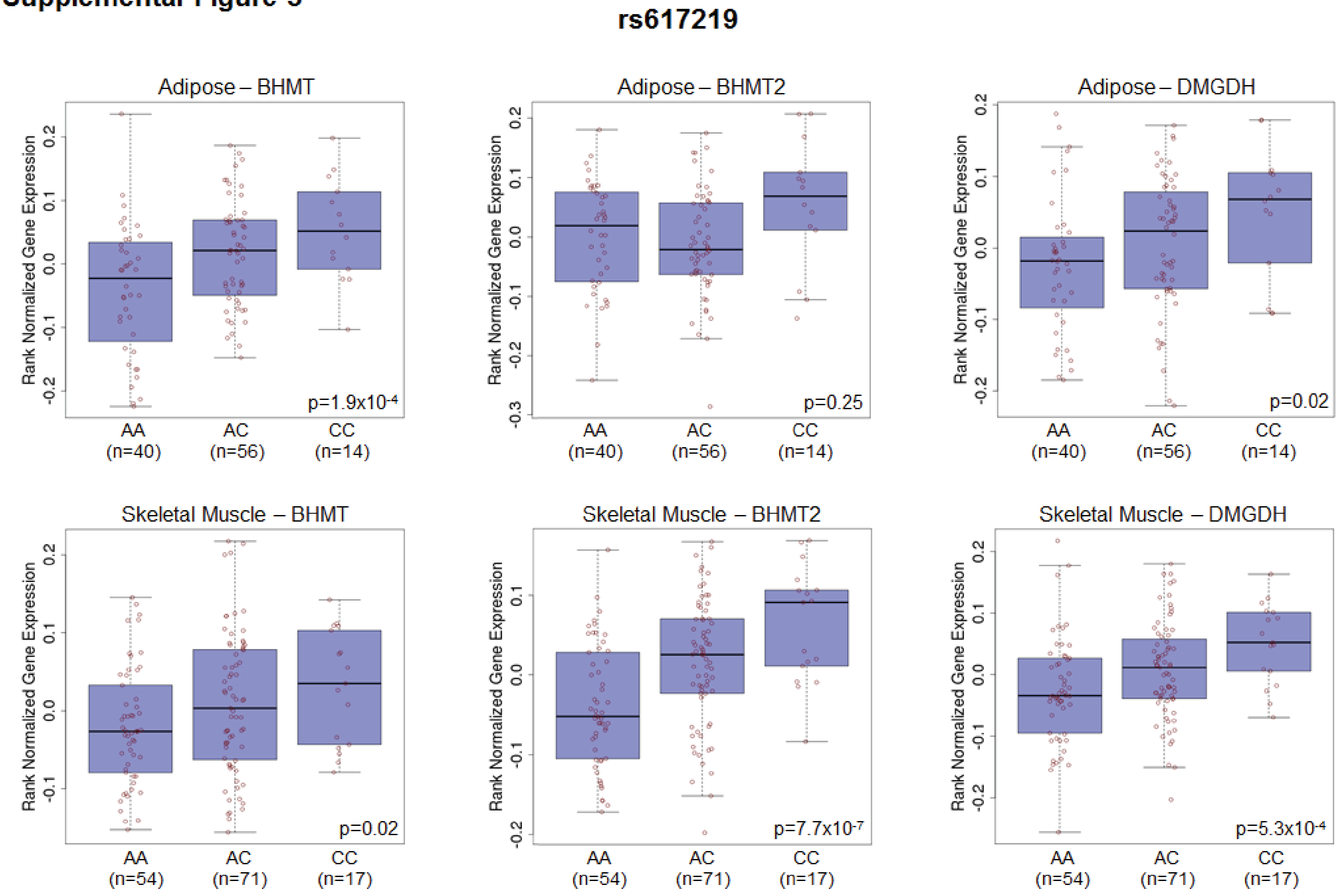

Supplemental Figure 3. ***Cis*** **eQTLs for** ***BHMT*****,** ***BHMT2*****, and** ***DMGDH*** **with rs617219 at the Chromosome 5q14.1 Locus.** Rs617219 yielded eQTLs for *BHMT*, *BHMT2*, or *DMGDH* at varying levels of significance in subcutaneous adipose tissue or skeletal muscle. Data were taken from the publicly available Genotype-Tissue Expression Project database (http://www.gtexportal.org/).

Supplemental Table 1. Multivariate Analyses of Chromosomes 2q34 Locus (rs715) with Risk of CAD in the GeneBank Cohort Stratified by Sex.

	Females		Males	
**Metabolite**	**OR (95% CI)**	**p-value**	**OR (95% CI)**	**p-value**
None	0.63 (0.40-0.98)	0.04	0.88 (0.59-1.32)	0.54
Betaine	0.57 (0.33-0.94)	0.03	0.90 (0.55-1.46)	0.66
Glycine	0.69 (0.43-1.11)	0.12	0.96 (0.64-1.46)	0.86
Betaine and Glycine	0.64 (0.37-1.11)	0.11	0.94 (0.57-1.54)	0.81

Multivariate logistic regression analyses were carried out in in the subset of 400 age-, sex-, and CAD-matched GeneBank subjects for whom genetic, metabolomic, and clinical phenotype data were all available. Odds ratios (OR) with 95% confidence intervals (CI) were calculated for the C allele of rs715, with inclusion of the indicated metabolite in the multivariate model.

Supplemental Table 2. Association of Chromosome 5q14.1 SNPs with Prevalent CAD in the CARDIoGRAM Consortium.

SNP	n	Effect Allele	Frequency	OR (95% CI)	p–value
rs16876394	81,108	C	0.14	1.00 (0.96-1.04)	1.0
rs557302	84,211	G	0.51	1.01 (0.99-1.04)	0.29
*rs567754	67,852	C	0.65	1.00 (0.97-1.03)	0.96

Analyses were adjusted for age and sex.
*Used as proxy for rs617219 ($r^2 = 0.84$).

Supplementary Note 1

Members of the CARDIoGRAM Consortium

Executive Committee: Sekar Kathiresan[1,2,3], Muredach P. Reilly[4], Nilesh J. Samani[5,6], Heribert Schunkert[7,79]

Executive Secretary: Jeanette Erdmann[7,79]

Steering Committee: Themistocles L. Assimes[8], Eric Boerwinkle[9], Jeanette Erdmann[7,79] Alistair Hall[10], Christian Hengstenberg[11], Sekar Kathiresan[1,2,3], Inke R. König[12], Reijo Laaksonen[13], Ruth McPherson[14], Muredach P. Reilly[4], Nilesh J. Samani[5,6], Heribert Schunkert[7,79], John R. Thompson[15], Unnur Thorsteinsdottir[16,17], Andreas Ziegler[12]

Statisticians: Inke R. König[12] (chair), John R. Thompson[15] (chair), Devin Absher[18], Li Chen[19], L. Adrienne Cupples[20,21], Eran Halperin[22], Mingyao Li[23], Kiran Musunuru[1,2,3], Michael Preuss[12,7], Arne Schillert[12], Gudmar Thorleifsson[16], Benjamin F. Voight[2,3,24], George A. Wells[25]

Writing group: Themistocles L. Assimes[8], Panos Deloukas[26], Jeanette Erdmann[7,79], Hilma Holm[16], Sekar Kathiresan[1,2,3], Inke R. König[12], Ruth McPherson[14], Muredach P. Reilly[4], Robert Roberts[14], Nilesh J. Samani[5,6], Heribert Schunkert[7,79], Alexandre F. R. Stewart[14]

ADVANCE: Devin Absher[18], Themistocles L. Assimes[8], Stephen Fortmann[8], Alan Go[27], Mark Hlatky[8], Carlos Iribarren[27], Joshua Knowles[8], Richard Myers[18], Thomas Quertermous[8], Steven Sidney[27], Neil Risch[28], Hua Tang[29]

CADomics: Stefan Blankenberg[30]*, Tanja Zeller[30]*, Arne Schillert[12], Philipp Wild[30], Andreas Ziegler[12], Renate Schnabel[30]*, Christoph Sinning[30]*, Karl Lackner[31], Laurence Tiret[32], Viviane Nicaud[32], Francois Cambien[32], Christoph Bickel[30], Hans J. Rupprecht[30], Claire Perret[32], Carole Proust[32], Thomas Münzel[30]

CHARGE: Maja Barbalic[33], Joshua Bis[34], Eric Boerwinkle[9], Ida Yii-Der Chen[35], L. Adrienne Cupples[20,21], Abbas Dehghan[36], Serkalem Demissie-Banjaw[37,21], Aaron Folsom[38], Nicole Glazer[39], Vilmundur Gudnason[40,41], Tamara Harris[42], Susan Heckbert[43], Daniel Levy[21], Thomas Lumley[44], Kristin Marciante[45], Alanna Morrison[46], Christopher J. O´Donnell[47], Bruce M. Psaty[48], Kenneth Rice[49], Jerome I. Rotter[35], David S. Siscovick[50], Nicholas Smith[43], Albert Smith[40,41], Kent D. Taylor[35], Cornelia van Duijn[36], Kelly Volcik[46], Jaqueline Whitteman[36], Vasan Ramachandran[51], Albert Hofman[36], Andre Uitterlinden[52,36]

deCODE: Solveig Gretarsdottir[16], Jeffrey R. Gulcher[16], Hilma Holm[16], Augustine Kong[16], Kari Stefansson[16,17], Gudmundur Thorgeirsson[53,17], Karl Andersen[53,17], Gudmar Thorleifsson[16], Unnur Thorsteinsdottir[16,17]

GERMIFS I and II: Jeanette Erdmann[7,79], Marcus Fischer[11], Anika Grosshennig[12,7], Christian Hengstenberg[11], Inke R. König[12], Wolfgang Lieb[54], Patrick Linsel-Nitschke[7], Michael Preuss[12,7], Klaus Stark[11], Stefan Schreiber[55], H.-Erich Wichmann[56,58,59], Andreas Ziegler[12], Heribert Schunkert[7,79]

GERMIFS III (KORA): Zouhair Aherrahrou[7,79], Petra Bruse[7,79], Angela Doering[56], Jeanette Erdmann[7,79], Christian Hengstenberg[11], Thomas Illig[56], Norman Klopp[56], Inke R. König[12], Patrick Diemert[7], Christina Loley[12,7], Anja Medack[7,79], Christina Meisinger[56], Thomas Meitinger[57,60], Janja Nahrstedt[12,7], Annette Peters[56], Michael Preuss[12,7], Klaus Stark[11], Arnika K. Wagner[7], H.-Erich Wichmann[56,58,59], Christina Willenborg[,7,79], Andreas Ziegler[12], Heribert Schunkert[7,79]

LURIC/AtheroRemo: Bernhard O. Böhm[61], Harald Dobnig[62], Tanja B. Grammer[63], Eran Halperin[22], Michael M. Hoffmann[64], Marcus Kleber[65], Reijo Laaksonen[13], Winfried März[63,66,67],

Andreas Meinitzer[66], Bernhard R. Winkelmann[68], Stefan Pilz[62], Wilfried Renner[66], Hubert Scharnagl[66], Tatjana Stojakovic[66], Andreas Tomaschitz[62], Karl Winkler[64]

MIGen: Benjamin F. Voight[2,3,24], Kiran Musunuru[1,2,3], Candace Guiducci[3], Noel Burtt[3], Stacey B. Gabriel[3], David S. Siscovick[50], Christopher J. O'Donnell[47], Roberto Elosua[69], Leena Peltonen[49], Veikko Salomaa[70], Stephen M. Schwartz[50], Olle Melander[26], David Altshuler[71,3], Sekar Kathiresan[1,2,3]

OHGS: Alexandre F. R. Stewart[14], Li Chen[19], Sonny Dandona[14], George A. Wells[25], Olga Jarinova[14], Ruth McPherson[14], Robert Roberts[14]

PennCATH/MedStar: Muredach P. Reilly[4], Mingyao Li[23], Liming Qu[23], Robert Wilensky[4], William Matthai[4], Hakon H. Hakonarson[72], Joe Devaney[73], Mary Susan Burnett[73], Augusto D. Pichard[73], Kenneth M. Kent[73], Lowell Satler[73], Joseph M. Lindsay[73], Ron Waksman[73], Christopher W. Knouff[74], Dawn M. Waterworth[74], Max C. Walker[74], Vincent Mooser[74], Stephen E. Epstein[73], Daniel J. Rader[75,4]

WTCCC: Nilesh J. Samani[5,6], John R. Thompson[15], Peter S. Braund[5], Christopher P. Nelson[5], Benjamin J. Wright[76], Anthony J. Balmforth[77], Stephen G. Ball[78], Alistair S. Hall[10], Wellcome Trust Case Control Consortium

Affiliations of CARDIoGRAM Consortium Members

1 Cardiovascular Research Center and Cardiology Division, Massachusetts General Hospital, Boston, MA, USA; 2 Center for Human Genetic Research, Massachusetts General Hospital, Boston, MA, USA; 3 Program in Medical and Population Genetics, Broad Institute of Harvard and Massachusetts Institute of Technology (MIT), Cambridge, MA, USA; 4 The Cardiovascular Institute, University of Pennsylvania, Philadelphia, PA, USA; 5 Department of Cardiovascular

Sciences, University of Leicester, Glenfield Hospital, Leicester, UK; 6 Leicester National Institute for Health Research Biomedical Research Unit in Cardiovascular Disease, Glenfield Hospital, Leicester, LE3 9QP, UK; 7 Medizinische Klinik II, Universität zu Lübeck, Lübeck, Germany; 8 Department of Medicine, Stanford University School of Medicine, Stanford, CA, USA; 9 University of Texas Health Science Center, Human Genetics Center and Institute of Molecular Medicine, Houston, TX, USA; 10 Division of Cardiovascular and Neuronal Remodelling, Multidisciplinary Cardiovascular Research Centre, Leeds Institute of Genetics, Health and Therapeutics, University of Leeds, UK; 11 Klinik und Poliklinik für Innere Medizin II, Universität Regensburg, Regensburg, Germany; 12 Institut für Medizinische Biometrie und Statistik, Universität zu Lübeck, Lübeck, Germany; 13 Science Center, Tampere University Hospital, Tampere, Finland; 14 The John & Jennifer Ruddy Canadian Cardiovascular Genetics Centre, University of Ottawa Heart Institute, Ottawa, Canada; 15 Department of Health Sciences, University of Leicester, Leicester, UK; 16 deCODE Genetics, 101 Reykjavik, Iceland; 17 University of Iceland, Faculty of Medicine, 101 Reykjavik, Iceland; 18 Hudson Alpha Institute, Huntsville, Alabama, USA; 19 Cardiovascular Research Methods Centre, University of Ottawa Heart Institute, 40 Ruskin Street, Ottawa, Ontario, Canada, K1Y 4W7; 20 Department of Biostatistics, Boston University School of Public Health, Boston, MA USA; 21 National Heart, Lung and Blood Institute's Framingham Heart Study, Framingham, MA, USA; 22 The Blavatnik School of Computer Science and the Department of Molecular Microbiology and Biotechnology, Tel-Aviv University, Tel-Aviv, Israel, and the International Computer Science Institute, Berkeley, CA, USA; 23 Biostatistics and Epidemiology, University of Pennsylvania, Philadelphia, PA, USA; 24 Department of Medicine, Harvard Medical School, Boston, MA, USA; 25 Research Methods, Univ Ottawa Heart Inst; 26 Department of Clinical Sciences,

Hypertension and Cardiovascular Diseases, Scania University Hospital, Lund University, Malmö, Sweden; 27 Division of Research, Kaiser Permanente, Oakland, CA, USA; 28 Institute for Human Genetics, University of California, San Francisco, San Francisco, CA, USA; 29 Dept Cardiovascular Medicine, Cleveland Clinic; 30 Medizinische Klinik und Poliklinik, Johannes-Gutenberg Universität Mainz, Universitätsmedizin, Mainz, Germany; 31 Institut für Klinische Chemie und Laboratoriumsmediizin, Johannes-Gutenberg Universität Mainz, Universitätsmedizin, Mainz, Germany; 32 INSERM UMRS 937, Pierre and Marie Curie University (UPMC, Paris 6) and Medical School, Paris, France; 33 University of Texas Health Science Center, Human Genetics Center, Houston, TX, USA; 34 Cardiovascular Health Resarch Unit and Department of Medicine, University of Washington, Seattle, WA USA; 35 Cedars-Sinai Medical Center, Medical Genetics Institute, Los Angeles, CA, USA; 36 Erasmus Medical Center, Department of Epidemiology, Rotterdam, The Netherlands; 37 Boston University, School of Public Health, Boston, MA, USA; 38 University of Minnesota School of Public Health, Division of Epidemiology and Community Health, School of Public Health (A.R.F.), Minneapolis, MN, USA; 39 University of Washington, Cardiovascular Health Research Unit and Department of Medicine, Seattle, WA, USA; 40 Icelandic Heart Association, Kopavogur Iceland; 41 University of Iceland, Reykjavik, Iceland; 42 Laboratory of Epidemiology, Demography, and Biometry, Intramural Research Program, National Institute on Aging, National Institutes of Health, Bethesda MD, USA; 43 University of Washington, Department of Epidemiology, Seattle, WA, USA; 44 University of Washington, Department of Biostatistics, Seattle, WA, USA; 45 University of Washington, Department of Internal Medicine, Seattle, WA, USA; 46 University of Texas, School of Public Health, Houston, TX, USA; 47 National Heart, Lung and Blood Institute, Framingham Heart Study, Framingham, MA and Cardiology Division,

Massachusetts General Hospital, Boston, MA, USA; 48 Center for Health Studies, Group Health, Departments of Medicine, Epidemiology, and Health Services, Seattle, WA, USA; 49 The Wellcome Trust Sanger Institute, The Wellcome Trust Genome Campus, Hinxton, Cambridge, UK; 50 Cardiovascular Health Research Unit, Departments of Medicine and Epidemiology, University of Washington, Seattle; 51 Boston University Medical Center, Boston, MA, USA; 52 Department of Internal Medicine, Erasmus Medical Center, Rotterdam, The Netherlands; 53 Department of Medicine, Landspitali University Hospital, 101 Reykjavik, Iceland; 54 Boston University School of Medicine, Framingham Heart Study, Framingham, MA, USA; 55 Institut für Klinische Molekularbiologie, Christian-Albrechts Universität, Kiel, Germany; 56 Institute of Epidemiology, Helmholtz Zentrum München – German Research Center for Environmental Health, Neuherberg, Germany; 57 Institut für Humangenetik, Helmholtz Zentrum München, Deutsches Forschungszentrum für Umwelt und Gesundheit, Neuherberg, Germany; 58 Institute of Medical Information Science, Biometry and Epidemiology, Ludwig-Maximilians-Universität München, Germany; 59 Klinikum Grosshadern, Munich, Germany; 60 Institut für Humangenetik, Technische Universität München, Germany; 61 Division of Endocrinology and Diabetes, Graduate School of Molecular Endocrinology and Diabetes, University of Ulm, Ulm, Germany; 62 Division of Endocrinology, Department of Medicine, Medical University of Graz, Austria; 63 Synlab Center of Laboratory Diagnostics Heidelberg, Heidelberg, Germany; 64 Division of Clinical Chemistry, Department of Medicine, Albert Ludwigs University, Freiburg, Germany; 65 LURIC non profit LLC, Freiburg, Germany; 66 Clinical Institute of Medical and Chemical Laboratory Diagnostics, Medical University Graz, Austria; 67 Institute of Public Health, Social and Preventive Medicine, Medical Faculty Manneim, University of Heidelberg, Germany; 68 Cardiology Group Frankfurt-Sachsenhausen, Frankfurt, Germany; 69

Cardiovascular Epidemiology and Genetics Group, Institut Municipal d'Investigació Mèdica, Barcelona; Ciber Epidemiología y Salud Pública (CIBERSP), Spain; 70 Chronic Disease Epidemiology and Prevention Unit, Department of Chronic Disease Prevention, National Institute for Health and Welfare, Helsinki, Finland; 71 Department of Molecular Biology and Center for Human Genetic Research, Massachusetts General Hospital, Harvard Medical School, Boston, USA; 72 The Center for Applied Genomics, Children's Hospital of Philadelphia, Philadelphia, Pennsylvania, USA; 73 Cardiovascular Research Institute, Medstar Health Research Institute, Washington Hospital Center, Washington, DC 20010, USA; 74 Genetics Division and Drug Discovery, GlaxoSmithKline, King of Prussia, Pennsylvania 19406, USA; 75 The Institute for Translational Medicine and Therapeutics, School of Medicine, University of Pennsylvania, Philadelphia, PA, USA; 76 Department of Cardiovascular Surgery, University of Leicester, Leicester, UK; 77 Division of Cardiovascular and Diabetes Research, Multidisciplinary Cardiovascular Research Centre, Leeds Institute of Genetics, Health and Therapeutics, University of Leeds, Leeds, LS2 9JT, UK; 78 LIGHT Research Institute, Faculty of Medicine and Health, University of Leeds, Leeds, UK; 79 Deutsches Zentrum für Herz-Kreislauf-Forschung (DZHK), Universität zu Lübeck, Lübeck, Germany

*Present Address: Department of General and Interventional Cardiology, University Heart Center Hamburg, Hamburg, Germany.

Sources of Funding for the CARDIoGRAM Consortium

The **ADVANCE** study was supported by a grant from the Reynold's Foundation and NHLBI grant HL087647.

Genetic analyses of **CADomics** were supported by a research grant from Boehringer Ingelheim. Recruitment and analysis of the CADomics cohort was supported by grants from Boehringer Ingelheim and PHILIPS medical Systems, by the Government of Rheinland-Pfalz in the context of the "Stiftung Rheinland-Pfalz für Innovation", the research program "Wissen schafft Zukunft" and by the Johannes-Gutenberg University of Mainz in the context of the "Schwerpunkt Vaskuläre Prävention" and the "MAIFOR grant", by grants from the Fondation de France, the French Ministry of Research, and the Institut National de la Santé et de la Recherche Médicale.

The **deCODE** CAD/MI Study was sponsored by NIH grant, National Heart, Lung and Blood Institute R01HL089650-02.

The German MI Family Studies (**GerMIFS I-III (KORA)**) were supported by the Deutsche Forschungsgemeinschaft and the German Federal Ministry of Education and Research (BMBF) in the context of the German National Genome Research Network (NGFN-2 and NGFN-plus), the EU funded integrated project Cardiogenics (LSHM-CT-2006-037593) and ENGAGE, and the bi-national BMBF/ANR funded project CARDomics (01KU0908A).

LURIC has received funding from the EU framework 6 funded Integrated Project "Bloodomics" (LSHM-CT-2004-503485), the EU framework 7 funded Integrated Project AtheroRemo (HEALTH-F2-2008-201668) and from Sanofi/Aventis, Roche, Dade Behring/Siemens, and AstraZeneca.

The **MIGen** study was funded by the US National Institutes of Health (NIH) and National Heart, Lung, and Blood Institute's STAMPEED genomics research program through R01 HL087676. Ron Do from the MIGen study is supported by a Canada Graduate Doctoral Scholarship from the Canadian Institutes of Health Research.

Recruitment of **PennCATH** was supported by the Cardiovascular Institute of the University of Pennsylvania. Recruitment of the **MedStar** sample was supported in part by the MedStar Research Institute and the Washington Hospital Center and a research grant from GlaxoSmithKline. Genotyping of PennCATH and Medstar was performed at the Center for Applied Genomics at the Children's Hospital of Philadelphia and supported by GlaxoSmithKline through an Alternate Drug Discovery Initiative research alliance award (M. P. R. and D. J. R.) with the University of Pennsylvania School of Medicine.

The **Ottawa Heart Genomic Study** was supported by CIHR #MOP--82810 (R. R.), CFI #11966 (R. R.), HSFO #NA6001 (R. McP.), CIHR #MOP172605 (R. McP.), CIHR #MOP77682 (A. F. R. S.).

The **WTCCC** Study was funded by the Wellcome Trust. Recruitment of cases for the WTCCC Study was carried out by the British Heart Foundation (BHF) Family Heart Study Research Group and supported by the BHF and the UK Medical Research Council. N. J. S. and S. G. B. hold chairs funded by the British Heart Foundation. N. J. S. and A.H.G are also supported by the Leicester NIHR Biomedical Research Unit in Cardiovascular Disease and the work described in this paper is part of the research portfolio of the Leicester NIHR Biomedical Research Unit.

The **Age, Gene/Environment Susceptibility Reykjavik Study** has been funded by NIH contract N01-AG-12100, the NIA Intramural Research Program, Hjartavernd (the Icelandic Heart Association), and the Althingi (the Icelandic Parliament).

The **Cleveland Clinic GeneBank** study was supported by NHLBI grants P01HL098055, P01HL076491, R01HL103866, P20HL113452, and R01HL103931.

The collection of clinical and sociodemographic data in the **Dortmund Health Study** was supported by the German Migraine- & Headache Society (DMKG) and by unrestricted grants of equal share from Astra Zeneca, Berlin Chemie, Boots Healthcare, Glaxo-Smith-Kline, McNeil Pharma (former Woelm Pharma), MSD Sharp & Dohme and Pfizer to the University of Muenster. Blood collection was done through funds from the Institute of Epidemiology and Social Medicine, University of Muenster.

The **EPIC-Norfolk study** is supported by the Medical Research Council UK and Cancer Research UK.

The **EpiDREAM study** is supported by the Canadian Institutes of Health Research, Heart and Stroke Foundation of Ontario, Sanofi-Aventis, GlaxoSmithKline and King Pharmaceuticals. Funding for Andrew Lotery from the **LEEDS** study was provided by the T.F.C. Frost charity and the Macular Disease Society.

The **Rotterdam Study** is supported by the Erasmus Medical Center and Erasmus University Rotterdam; the Netherlands Organization for Scientific Research; the Netherlands Organization for Health Research and Development (ZonMw); the Research Institute for Diseases in the Elderly; The Netherlands Heart Foundation; the Ministry of Education, Culture and Science; the Ministry of Health Welfare and Sports; the European Commission (DG XII); and the Municipality of Rotterdam. Support for genotyping was provided by the Netherlands Organization for Scientific Research (NWO) (175.010.2005.011, 911.03.012), the Netherlands Genomics Initiative (NGI)/ NWO project nr. 050-060-810 and Research Institute for Diseases in the Elderly (RIDE). Abbas Dehghan is supported by a grant from NWO (Vici, 918-76-619).

The **SAS** study was funded by the British Heart Foundation.

The Swedish Research Council, the Swedish Heart & Lung Foundation and the Stockholm County Council (ALF) supported the **SHEEP** study.

SMILE was funded by the Netherlands Heart foundation (NHS 92345). Dr Rosendaal is a recipient of the Spinoza Award of the Netherlands Organisation for Scientific Research (NWO) which was used for part of this work.

The **Verona Heart Study** was funded by grants from the Italian Ministry of University and Research, the Veneto Region, and the Cariverona Foundation, Verona.

The **Atherosclerosis Risk in Communities Study** is carried out as a collaborative study supported by National Heart, Lung, and Blood Institute contracts N01-HC-55015, N01-HC-55016, N01-HC-55018, N01-HC-55019, N01-HC-55020, N01-HC-55021, and N01-HC-55022. The authors thank the staff and participants of the ARIC study for their important contributions.

The **KORA** (Kooperative Gesundheitsforschung in der Region Augsburg) research platform was initiated and financed by the Helmholtz Zentrum München - National Research Center for Environmental Health, which is funded by the German Federal Ministry of Education, Science, Research and Technology and by the State of Bavaria. Part of this work was financed by the German National Genome Research Network (NGFN-2 and NGFNPlus) and within the Munich Center of Health Sciences (MC Health) as part of LMUinnovativ.

Work described in this paper is part of the research portfolio supported by the Leicester NIHR Biomedical Research Unit in Cardiovascular Disease.

This work forms part of the research themes contributing to the translational research portfolio of Barts and the London Cardiovascular Biomedical Research Unit which is supported and funded by the National Institute of Health Research.
